# Hyperosmolarity-Induced Oxidative Stress Leads to Senescence in Human Corneal Epithelial Cells (HCEPC) via DNA Damage, Metabolic Disturbance and Mitophagy Decline

**DOI:** 10.3390/antiox14111381

**Published:** 2025-11-19

**Authors:** Yongjie Zhang, Tingjun Fan

**Affiliations:** Key Laboratory for Corneal Tissue Engineering, College of Marine Life Sciences, Ocean University of China, Qingdao 266003, China

**Keywords:** hyperosmolarity, oxidative stress, cell senescence, autophagy

## Abstract

Background: Dry eye disease (DED), characterized by tear film hyperosmolarity, can lead to corneal epithelial damage. The mechanisms linking hyperosmotic stress to human corneal epithelial cell (HCEPC) damage are not fully understood. Methods: A DED model was established by exposing HCEPCs to sustained hyperosmotic stress (400 mOsm/L) over multiple passages in vitro. Senescence was assessed using senescence-associated-β-galactosidase (SA-β-gal) staining, 5-ethynyl-2′-deoxyuridine (EdU) assays, p16^INK4A^ and senescence-associated secretory phenotypes (SASP) analysis. Mechanisms were investigated by measuring reactive oxygen species (ROS), mitochondrial function, energy metabolism, DNA damage, and inflammatory signaling. The role of autophagy was probed pharmacologically. Results: Hyperosmotic stress induced HCEPC senescence, driven by mitochondrial dysfunction, oxidative stress, DNA damage, bioenergetic crisis, and compromised autophagy (especially mitophagy). Autophagy and mitophagy play a key role in regulating senescence progression. Enhancing autophagy with LYN-1604 ameliorated oxidative stress, improved energy homeostasis, and attenuated senescence. Inhibiting autophagy exacerbated these states. Conclusion: Hyperosmolarity promotes HCEPC senescence via mitochondrial dysfunction and oxidative damage. Autophagy serves a critical protective role, and its enhancement represents a promising therapeutic strategy for DED.

## 1. Introduction

Dry eye disease (DED) is a multifactorial chronic inflammatory condition and one of the most prevalent ocular disorders worldwide [1]. It is characterized by loss of tear film homeostasis, accompanied by ocular symptoms such as tear fluid hyperosmolarity, ocular surface inflammation and damage, and neurosensory abnormalities [2]. Mild cases may cause dryness, prickling, and blurred vision, while severe cases can lead to corneal perforation resulting in blindness eventually [3]. Numerous risk factors contribute to DED, including autoimmune diseases, endocrine dysfunction, ocular infections, environmental insults, postoperative complications, and medication side effects [4]. Current global prevalence rates range between 5.5% and 33.7% [5], with estimated rates of 21–30% in China [6]—numbers that continue to rise due to increased screen time, environmental pollution, and an aging population [7]. DED has become the most frequently occurring ocular disease, increasingly affecting younger demographics, posing a substantial threat to public visual health and quality of life [8]. There is thus an urgent need to elucidate the pathogenic mechanisms of DED to establish a theoretical foundation for its prevention and treatment.

DED is primarily classified into two subtypes, aqueous-deficient and evaporative, with the latter accounting for over 80% of cases, which result in hyperosmolarity in the tear, the main characteristic of DED [9]. Under physiological conditions, tear osmolarity is approximately 312 mOsm/L [10]. In DED patients, however, it can rise as high as 360–424 mOsm/L [11,12]. Current research indicates that excessive reactive oxygen species (ROS) production induced by hyperosmotic stress is a central mechanism underlying corneal damage in DED [13]. Elevated ROS levels cause oxidative stress, which is heavily implicated in disease progression. Under normal conditions, endogenous antioxidant systems maintain ROS below pathological thresholds [14]. An imbalance between ROS generation and antioxidant capacity leads to oxidative stress, inflammation, tear film instability, and reduced tear secretion [13,15]. Additionally, mitochondrial oxidative damage is closely associated with lacrimal gland dysfunction: ROS accumulation within mitochondria promotes carbonylated protein aggregation in glandular tissues, triggering inflammation and fibrosis around acinar cells and ultimately contributing to DED [16]. A study by Deng et al. [17] utilizing hyperosmotic culture media to simulate elevated tear fluid osmolarity has demonstrated that such conditions induce oxidative damage in human corneal epithelial cells (HCEPC) in vitro. This is characterized by increased generation of ROS and a disruption in the balance between pro-oxidant and antioxidant enzymes, ultimately leading to mitochondrial DNA damage and peroxidation of cell membrane lipids [17].

In general, hyperosmotic stress in DED elevates intracellular levels of ROS. Our previous work has demonstrated that sustained oxidative stress can trigger cellular senescence [18,19]. However, the precise mechanisms underlying DED-induced corneal damage and its impact on cell fate decisions remain incompletely understood. Therefore, this study aims to elucidate the pathogenic mechanisms and molecular basis of DED, as well as to strategically target its key regulatory hubs, which could pave the way for novel therapeutic interventions.

## 2. Materials and Methods

### 2.1. HCEP Cell Culture and Experimental Design

As previously described [20,21], HCEPCs at passage 53 for initiating experiments, derived from a continuous non-transfected HCEP cell line established in our laboratory [22], were cultured in Dulbecco’s Modified Eagle Medium (DMEM)-F12 medium (10-092-CVR, Corning, Corning, NY, USA) supplemented with 10% (*v*/*v*) fetal bovine serum (FBS; 10100147, Gibco, Grand Island, NY,, USA) and maintained in a humidified incubator at 37 °C with 5% CO_2_.

The experiment was designed as follows: (i) hyperosmotic medium (400 mOsm/L) was prepared by supplementing DMEM-F12 culture (312 mOsm/L) with an additional 2.92 g/L of NaCl, and its osmolarity was measured and confirmed using a molarity os-mometer (SMC 30DS, Tianhe, Tianjin, China); (ii) the HCEPCs from a the non-transfected HCEP cell line were trypsinized, resuspended in hyperosmotic medium, seeded into cell culture plates or flasks, collected as the cell confluent monolayer formed and divided into 2 parts: one was cultured in the normal 10% FBS-DMEM/F12 for 24 h allowing stress re-covery before senescence assessment and designated as the first hyperosmotic passage (hs-P1), the other was cultured in the hyperosmotic media for successive induction of se-nescence as hs-P2 or hs-P3; (iii) to explore the role of autophagy in modulating HCEPC senescence, we prepared the autophagy inhibitor SBI-0206965 (5 μM; HY-16966, Med-Chemexpress, Monmouth Junction, NJ, USA) [23] or activator LYN-1604 (2 μM; HY-101923 MedChemexpress, NJ, USA) [24]; (iv) the inhibitor or activator was applied simultaneously with hyperos-motic stress induction and maintained continuously throughout the experimental dura-tion, generating treatment groups designated as hs + SBI-0206965 and hs + LYN-1604; (v) correspondingly, the HCEPCs cultured in the DMEM-F12 without hyperosmotic stress was used as the control group (Ctrl).

### 2.2. Microscopy Observation

HCEPCs were cultured before a confluent monolayer was achieved under standard conditions (37 °C, 5% CO_2_). The duration of cell growth was recorded, and cellular morphology was observed using an inverted microscope (TS100, Nikon, Tokyo, Japan). Three 100× fields of view were randomly selected for photography from each cell sample. The area of each cell within these fields was measured using the “area measurement” function of the ImageJ software (Version 1.53 k, NIH, Bethesda, MD, USA).

### 2.3. Senescence-Associated-β-Galactosidase (SA-β-Gal) Detection

HCEPCs were seeded into 24-well plates and cultured under standard conditions. Cellular senescence was assessed using an SA-β-gal staining kit (C0602, Beyotime, Shanghai, China) according to the manufacturer’s instructions. Stained cells were visualized under an inverted microscope (TS100, Nikon, Tokyo, Japan). Three 100× fields of view were randomly selected for photography from each cell sample. Positively stained cells (blue-stained) and total cell counts were systematically quantified using the “Multi-point Tool” in ImageJ software (Version 1.53 k, NIH, USA). The percentage of SA-β-gal-positive cells was calculated as follows:

SA-β-Gal^+^% = (Number of SA-β-Gal-positive cells/Total number of cells) × 100% (1)

### 2.4. Cell Viability and Proliferation

Cell viability was evaluated using a Cell Counting Kit-8 (CCK-8; C0037, Beyotime, Shanghai, China) according to the manufacturer’s protocol. Absorbance was measured at 450 nm using a microplate reader (Multiskan GO, Thermo Fisher, Waltham, MA, USA), and viability was normalized to that of untreated controls. The CCK-8 assay measures mitochondrial dehydrogenase activity and reflects overall metabolic capacity to evaluate the gradual loss of metabolic activity and reduction in cell viability under sustained hyperosmotic stress.

Cell proliferation was assessed using an 5-ethynyl-2′-deoxyuridine (EdU) staining kit (C0071S; Beyotime, Shanghai, China). As per the manufacturer’s instructions, cell nuclei were counterstained with Hoechst 33342 provided in the kit to define cell presence. Three random fields per sample were captured using a 200× fluorescence microscope (E80i, Nikon, Tokyo, Japan). Image processing was performed using ImageJ software (Version 1.53 k, NIH, USA) through the following standardized protocol: first, the “Threshold” function was applied to convert fluorescent images into binary black-and-white images based on automated pixel intensity threshold; subsequently, the “Analyze Particles” module was employed to objectively quantify both EdU-positive cells and total cell counts per field. The percentage of EdU-positive cells was then calculated accordingly. The percentage of EdU-positive cells was calculated as:

EdU^+^% = (Number of EdU-positive cells/Total number of cells) × 100%. (2)

### 2.5. Immunofluorescence Staining

The cells were fixed with 4% paraformaldehyde (P1110, Solarbio, Beijing, China) for 15 min and permeabilized with 0.5% Triton X-100 (T8200, Solarbio, Beijing, China) in phosphate-buffered saline (PBS) for 10 min at 25 °C. After blocking with 2% bovine serum albumin (BSA; BS114, Biosharp, Hefei, China) for 30 min, the samples were incubated with primary antibodies for 2 h at room temperature, followed by three washes with PBS. Subsequently, the samples were incubated with appropriate secondary antibodies for 1 h at room temperature. Nuclei were stained with 4′,6-diamidino-2-phenylindole (DAPI; C1006, Beyotime, Shanghai, China), and after thorough washing with PBS. The primary antibodiy of γ-H2AX (1:500; ab303656) was purchased from Abcam (Cambridge, UK). The secondary antibodiy, Alexa Fluor 488 AffiniPure goat anti-rabbit (1:500; RGAR002), was purchased from Proteintech Biotechnology (Chicago, IL, USA). Images of three random fields per sample were captured at 200× magnification using a fluorescence microscope (E80i, Nikon, Tokyo, Japan). Image processing was performed using ImageJ software (Version 1.53 k, NIH, USA) through the following standardized protocol: first, the “Threshold” function was applied to convert fluorescent images into binary black-and-white images based on automated pixel intensity threshold; subsequently, the “Analyze Particles” module was employed to objectively quantify both γ-H2AX -positive cells and total cell counts per field. The percentage of γ-H2AX -positive cells was then calculated accordingly. The percentage of γ-H2AX -positive cells was calculated as:

γ-H2AX^+^% = (Number of γ-H2AX-positive cells/Total number of cells) × 100%. (3)

### 2.6. Western Blot

Total cellular proteins were extracted using radio-immunoprecipitation assay lysis buffer (RIPA) lysis buffer (R493085-01, Aladdin, Shanghai, China) containing protease (ST505, Beyotime, Shanghai, China) and phosphatase inhibitors (P885775, Macklin, Shanghai, China). The proteins were separated by sodium dodecyl sulfate-polyacrylamide gel electrophoresis (SDS-PAGE) and transferred onto polyvinylidene difluoride (PVDF) membranes (IPVH00010, Millipore, Bedford, MA, USA). The membranes were blocked with 5% skim milk (D8340, Solarbio, Beijing, China) and then incubated with primary antibodies overnight at 4 °C. After washing with phosphate-buffered saline with Tween 20 (PBST), the membranes were incubated with secondary antibodies for 2 h. Protein bands were visualized using an enhanced chemiluminescence (ECL) substrate kit (SQ201, Epizyme, Shanghai, China) and imaged with an Imaging System (Tannon, Beijing, China). The band optic intensities were quantified using ImageJ software. The primary antibodies were purchased from Proteintech Biotechnology (Chicago, IL, USA) as follows: p16-INK4A (1:1000; 10883-1-AP), p21 (1:1000, 10355-1-AP), nuclear factor kappa-light-chain-enhancer of activated B cells (NF-κB; 1:1000; 66535-1-Ig); cyclic GMP-AMP synthase (cGAS, 1:1000; 68640-1-Ig), stimulator of interferon genes (STING; 1:20,000; 19851-1-AP), phosphorylated adenosine monophosphate-activated protein kinase (p-AMPK; 1:100; 80209-6-RR), AMPK (1:2000; 10929-2-AP), mammalian target of rapamycin complex (mTOR; 1:5000; 66888-1-Ig), silent information regulator factor 2-related enzyme 1 (Sirt1; 1:1000; 13161-1-AP), nicotinamide phosphoribosyl transferase (NAMPT; 1:2000; 11776-1-AP), protein kinase R like endoplasmic reticulum kinase (PERK; 1:500; 24390-1-AP), p-PERK (1:500; 29546-1-AP), activating transcription factor 4 (ATF4; 1:500; 10835-1-AP), microtubule-associated protein 1 light chain 3 (LC3; 1:2000; 14600-1-AP), p62 (1:5000; 18420-1-AP), Unc-51 like autophagy activating kinase 1 (ULK1; 1:500; 20986-1-AP), PTEN-induced kinase 1 (PINK; 1:1000; 23274-1-AP), Nip3-like protein X (NIX; 1:500; 12986-1-AP), glyceraldehyde-3-phosphate dehydrogenase (GAPDH; 1:20,000; 60004-1-Ig), and β-actin (1:20,000; 66009-1-Ig). The secondary antibodies, HRP-Goat Anti-Rabbit (1:10,000; RGAR001) and HRP-Goat Anti-Mouse (1:10,000; RGAM001), were purchased from Proteintech Biotechnology (Chicago, IL, USA).

### 2.7. Enzyme-Linked Immunosorbent Assay (ELISA)

We employed the commercial ELISA kits for Interleukin (IL)-1β (JL13662, Jonlnbio, Shanghai, China), IL-6 (JL14113, Jonlnbio, Shanghai, China), and IL-8 (JL19291, Jonlnbio, Shanghai, China) measuring the IL-1β/6/8 levels in the cell culture supernatants according to the manufacturer’s instructions to evaluate changes in senescence-associated secretory phenotypes (SASP) secretion.

### 2.8. ROS Determination

The ROS were determined by incubating the HCEPCs with 10 μM DCFH-DA (in DMEM/F12; S0033S, Beyotime, Shanghai, China) for 40 min according to the manufacturer’s protocol. Three randomly selected fields of each sample were observed and imaged under a 200× fluorescence microscope (E80i, Nikon, Tokyo, Japan). The captured images were then converted into thresholded binary images by automatically setting the pixel intensity threshold with the “Threshold” function in ImageJ software (Version 1.53 k, NIH, USA). The mean fluorescence intensity of each image was subsequently measured using the “Measurement” tool.

### 2.9. JC-1 Staining

Mitochondrial membrane potential was assessed using the JC-1 mitochondrial membrane potential assay kit (C2003S, Beyotime, Shanghai, China) according to the manufacturer’s protocol. Fluorescence signals were detected using a fluorescence plate reader (Infinite M200Pro, TECAN, Männedorf, Switzerland). The mitochondrial membrane potential was expressed as the ratio of aggregated JC-1 (excitation/emission = 580/590 nm) to monomeric JC-1 (excitation/emission = 514/529 nm). Simultaneously, fluorescence micrographs of both the red channel (aggregated JC-1) and green channel (monomeric JC-1) were acquired from three random fields of view per cell sample using a 200× fluorescence microscope (E80i, Nikon, Tokyo, Japan) to visually assess the mitochondrial activity.

### 2.10. ADP/ATP Ratio Assay

The ADP/ATP ratio was evaluated using a commercial ADP/ATP ratio assay kit (MAK135, Sigma-Aldrich, St. Louis, MO, USA) following the manufacturer’s instructions and measured with a fluorescence plate reader (Infinite M200Pro, TECAN, Männedorf, Switzerland).

### 2.11. Nicotinamide Adenine Dinucleotide (NAD^+^) Level and NAD^+^/NADH Ratio Assay

Intracellular NAD^+^ concentration and the NAD^+^/NADH ratio were measured using an NAD^+^/NADH assay kit (S0175, Beyotime, Shanghai, China) with a microplate reader (Multiskan GO, Thermo Fisher, MA, USA), in accordance with the manufacturer’s standard protocol.

### 2.12. Glucose Uptake, Consumption and Lactic Acid Excretion

Glucose uptake tests were conducted and measured using a 2-NBDG fluorescence assay (HY-116215, MedChemexpress, NJ, USA) according to previous reports [25]. The glucose consumption experiment was conducted according to previous methods [26] and measured using a glucose consumption detection kit (S0201S, Beyotime, Shanghai, China). The lactate release experiment was conducted according to previous methods [27] and lactate was detected using an Assay Kit (ab65330, Abcam, Cambridge, UK).

### 2.13. Real-Time Reverse Transcription Polymerase Chain Reaction (RT-qPCR)

Total RNA was extracted from HCEPCs using a total RNA extraction kit (DP419, Tiangen, Beijing, China). RT-qPCR was subsequently performed using the One Step RT-PCR Kit (RR066A, Takara, Shiga, Japan) on a Roche Light Cycler 480 Real-Time PCR System (Roche, Basel, Switzerland), in accordance with the manufacturers’ standard protocols. The expression levels of the following autophagy-related genes were analyzed: Autophagy-related gene (*ATG*) 1 (NM_003565.4, forward: 5′-CCAGAGCAACATGATGGCG-3′, reverse: 5′-CCTTCCCGTCGTAGTGCTG-3′), *ATG6* (NM_017749.3, forward: 5′-GGTGTCTCTCGCAGATTCATC-3′, reverse: 5′-TCAGTCTTCGGCTGAGGTTCT-3′), *ATG7* (NM_001144912.2, forward: 5′-CAGTTTGCCCCTTTTAGTAGTGC-3′, reverse: 5′-CCAGCCGATACTCGTTCAGC-3′), and *ATG12* (NM_004707.4, forward: 5′-TAGAGCGAACACGAACCATCC-3′, reverse: 5′-CACTGCCAAAACACTCATAGAGA-3′). *β-actin* (NM_001101.5, forward: 5′-GAAGGTGACAGCAGTCGGTT-3′, reverse: 5′-AGTGGGGTGGCTTTTAGGAT-3′) was used as an internal reference gene for normalization.

### 2.14. Monodansylcadaverine (MDC) Staining

Autophagic activity was evaluated using an MDC staining kit (G0170, Solarbio, Beijing, China) according to the manufacturer’s instructions. Three randomly selected fields of each sample were observed and imaged under a 200× fluorescence microscope (E80i, Nikon, Tokyo, Japan). The captured images were then converted into thresholded binary images by automatically setting the pixel intensity threshold with the “Threshold” function in ImageJ software (Version 1.53 k, NIH, USA). The mean fluorescence intensity of each image was subsequently measured using the “Measurement” tool.

### 2.15. Janus Green B Staining

To visualize mitochondria, HCEPCs were stained with Janus Green B solution (G1571, Solarbio, Beijing, China) for 10 min at 37 °C. After washing with Ringer’s solution, three random fields per cell sample were recorded at both 400× and 1000× magnifications. The mitochondrial granules within cells under the 1000× fields were quantified using the “Multi-point Tool” in ImageJ software (Version 1.53 k, NIH, USA).

### 2.16. Mitophagy Assay

Mitophagy was assessed by co-staining mitochondria, lysosomes, and autophagosomes using MitoTracker Green (C1048, Beyotime, Shanghai, China), LysoTracker Red (C1046, Beyotime, Shanghai, China), and MDC, respectively, following the manufacturers’ protocols. The co-localization in three randomly selected fields of view for each cell sample was observed and imaged under a 1000× confocal laser scanning microscope (TCS SP8, Leica, Veszprém, Germany). The positive spots within cells under the 1000× fields were quantified using the “Multi-point Tool” in ImageJ software (Version 1.53 k, NIH, USA).

### 2.17. Statistical Analysis

Data are expressed as mean ± standard error of the mean (SEM). The mean value of triplicate experiments was calculated. Statistical significance was analyzed using one-way analysis of variance, followed by Tukey’s post hoc test for multiple comparisons. *p* < 0.05 was considered statistically significant; *p* < 0.01 was considered highly statistically significant.

## 3. Results

### 3.1. Establishment of a Dry Eye Disease Cellular Model Using Hyperosmotic Stress-Treated HCEPCs and Induction of HCEPC Senescence

**Figure 1:**
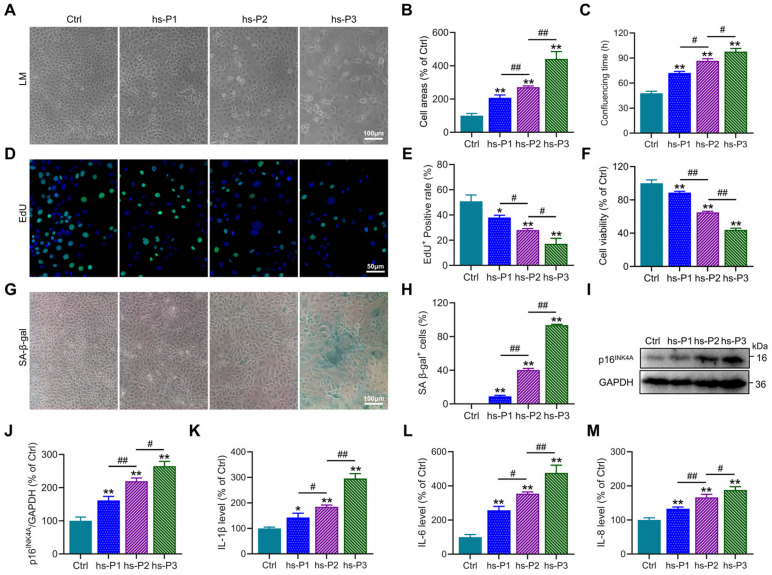
Hyperosmotic stress (400 mOsm/L) induces cellular senescence in HCEPCs. (**A**) Light microscopic images of HCEPCs treated with 400 mOsm/L hypertonic culture medium from hs-P1 to hs-P3 compared to the Ctrl. (**B**) Statistical analysis of cell area in A. (**C**) Statistical analysis of confluencing time in HCEPCs from hs-P1 to hs-P3 compared to the Ctrl. (**D**,**E**) EdU staining images and statistical analysis in HCEPCs from hs-P1 to hs-P3 compared to the Ctrl. Representative micrographs display EdU-positive cells (green) and total cells (blue). (**F**) Analysis of cell viability in HCEPCs from hs-P1 to hs-P3 compared to the Ctrl. (**G**,**H**) SA-β-gal staining images and quantitative analysis in HCEPCs from hs-P1 to hs-P3 compared to the Ctrl. (**I**,**J**) Western blot images and optical density analysis of p16^INK4A^ expression in HCEPCs from hs-P1 to hs-P3 compared to the Ctrl. (**K**–**M**) Secretion levels of IL-1β, IL-6, and IL-8 in HCEPCs from hs-P1 to hs-P3 compared to the Ctrl. hs-P denotes the number of successive passages under 400 mOsm/L hyperosmotic medium. Data are expressed as mean ± SEM (*n* = 3). * p < 0.05, ** p < 0.01 vs. blank controls (Ctrl), and *^#^ p* < 0.01, *^##^ p* < 0.01 among different experimental groups.

Establishment of a DED model in vitro was achieved by maintaining HCEPCs in 400 mOsm/L medium supplemented with NaCl over three consecutive passages to ensure sustained hyperosmotic stress. Preliminary observations revealed a progressive increase in cell surface area (Figure 1A,B), the time required to form confluent monolayers (Figure 1C), and the proportion of SA-β-gal-positive cells (Figure 1G,H) with successive passages under hyperosmotic conditions. Concurrently, a gradual decline in both proliferative capacity (Figure 1D,E) and cell viability (Figure 1F) was observed. These morphological and functional alterations collectively suggested the emergence of a senescent phenotype in HCEPCs under prolonged hyperosmotic exposure. To further corroborate cellular senescence, Western blot analysis was performed to assess the expression of the senescence-associated marker p16^INK4A^(Figure 1I) [28]. A consistent upregulation of p16^INK4A^ protein levels was detected (Figure 1J), indicative of cell cycle arrest. In addition, the secretion of SASP factors [29], including IL-1β, IL-6, and IL-8, was found to increase progressively with successive passages in the hyperosmotic environment (Figure 1K–M). In summary, these results demonstrate that sustained exposure to 400 mOsm/L hyperosmotic stress effectively induces senescence in HCEPCs.

### 3.2. Hyperosmotic Stress Mediates the Induction of Chromatin Damage and Subsequent Inflammatory Activation in HCEPCs

**Figure 2:**
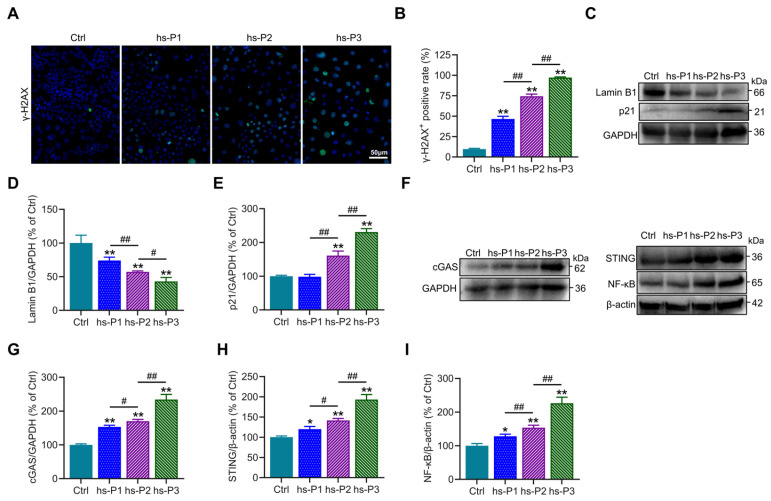
Hyperosmotic stress induces DNA damage and inflammatory response in HCEPCs. (**A**,**B**) Immunofluorescence staining and statistical analysis of γ-H2AX in HCEPCs from hs-P1 to hs-P3 compared to the Ctrl. Representative micrographs display γ-H2AX-positive cells (green) and total cells (blue). (**C**–**E**) Western blot images and optical density analysis of Lamin B1 and p21 expression in HCEPCs from hs-P1 to hs-P3 compared to the Ctrl. (**F**–**I**) Western blot images and optical density analysis of cGAS, STING, and NF-κB expression in HCEPCs from hs-P1 to hs-P3 compared to the Ctrl. hs-P denotes the number of successive passages under 400 mOsm/L hyperosmotic medium. Data are expressed as mean ± SEM (*n* = 3). * p < 0.05, ** p < 0.01 vs. blank controls (Ctrl), and *^#^ p* < 0.01, *^##^ p* < 0.01 among different experimental groups.

To further investigate the DNA damage effects of hyperosmotic stress on HCEPCs, immunofluorescence staining of the DNA damage marker γ-H2AX was performed, revealing a significant increase in the proportion of γ-H2AX-positive cells (Figure 2A,B). Concurrently, Western blot analysis showed a marked reduction in the expression of Lamin B1, a core component of the nuclear lamina, along with a gradual upregulation of p21 (Figure 2C–E). These findings indicate that hyperosmotic stress compromises nuclear envelope integrity and induces chromatin damage in HCEPCs, thereby initiating DNA damage response and upregulates p21, leading to cell cycle arrest and ultimately promoting cellular senescence. Further immunoblotting analyses showed that hyperosmotic exposure significantly upregulated the expression of key signaling molecules, including cGAS, STING, and NF-κB (Figure 2F–I), accompanied by markedly enhanced SASP factors secretion (Figure 1K–M). Taken together, these data suggest that hyperosmotic stress triggers nuclear damage in HCEPCs, leading to the leakage of DNA into the cytoplasm, which in turn activates the cGAS–STING–NF-κB signaling axis [30]. This cascade promotes inflammatory responses and SASP factors secretion, thereby creating a feed-forward loop that exacerbates cellular senescence [31].

### 3.3. Hyperosmotic Stress Causes Oxidative Stress in HCEPCs, Leading to Energy Stress Response and Unfolded Protein Response (UPR)

**Figure 3:**
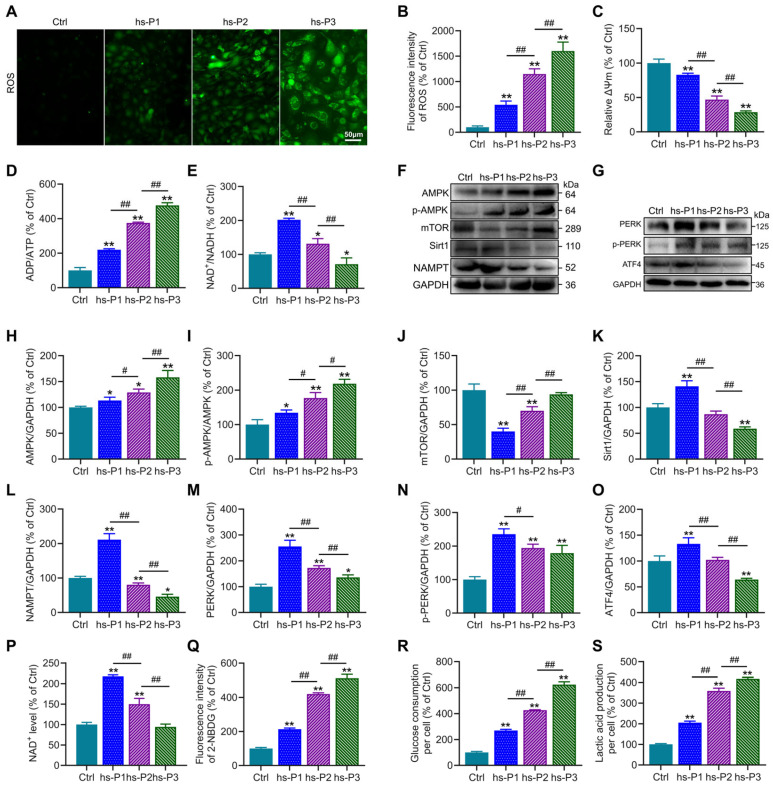
Hyperosmotic stress triggers oxidative stress-mediated energy crisis and UPR in HCEPCs. (**A**) ROS staining in HCEPCs from hs-P1 to hs-P3. (**B**) Statistical analysis of ROS levels in A. (**C**) Statistical analysis of mitochondrial membrane potential. (**D**) ADP/ATP ratio in HCEPCs under hyperosmotic stress from hs-P1 to hs-P3. (**E**) NAD^+^/NADH ratio in HCEPCs under hyperosmotic stress from hs-P1 to hs-P3. (**F**) Western blot images of p-AMPK, AMPK, mTOR, Sirt1, and NAMPT expression in HCEPCs from hs-P1 to hs-P3. (**G**) Western blot images of PERK, p-PERK, and ATF4 in HCEPCs from hs-P1 to hs-P3. (**H**–**L**) Optical density analysis of p-AMPK, AMPK, mTOR, Sirt1, and NAMPT in (**F**). (**M**–**O**) Optical density analysis of PERK, p-PERK, and ATF4 in (**G**). (**P**) Statistical analysis of intracellular NAD^+^ levels in HCEPCs from hs-P1 to hs-P3. (**Q**) Statistical analysis of glucose uptake in HCEPCs from hs-P1 to hs-P3. (**R**) Statistical analysis of glucose consumption in HCEPCs from hs-P1 to hs-P3. (**S**) Statistical analysis of lactic acid production in HCEPCs from hs-P1 to hs-P3. hs-P denotes the number of successive passages under 400 mOsm/L hyperosmotic medium. Data are expressed as mean ± SEM (*n* = 3). * p < 0.05, ** p < 0.01 vs. blank controls (Ctrl), and *^#^ p* < 0.01, *^##^ p* < 0.01 among different experimental groups.

To further investigate the mechanisms underlying hyperosmotic stress-induced senescence in HCEPCs, we first assessed ROS levels in HCEPCs subjected to hyperosmotic stress across hs-P1-3. Results demonstrated a significant, passage-dependent increase in ROS levels in hs-P1 to hs-P3 HCEPCs compared to Ctrl group (Figure 3A,B), suggesting that hyperosmotic stress-induced senescence may be associated with oxidative stress. To investigate the intracellular source of ROS, mitochondrial membrane potential (ΔΨm) was evaluated using JC-1 staining. This analysis revealed a progressive decline in ΔΨm with successive passages under hyperosmotic stress (Figure 3C and Figure S1), demonstrating that loss of ΔΨm and disruption of the electron transport chain constitute a significant source of elevated intracellular ROS. Mitochondrial dysfunction is frequently accompanied by impaired aerobic respiration and cellular energy deficits [32]. Consistent with this conjecture, we observed a progressive increase in the ADP/ATP ratio (Figure 3D) and a sustained decline in the NAD^+^/NADH ratio following an initial peak in hs-P1 (Figure 3E). Concomitant with rising ADP levels in hyperosmotic stress-treated HCEPCs, the expression of p-AMPK and total AMPK—key energy-sensing and metabolic regulatory kinases—was significantly elevated (Figure 3F,H,I). This was accompanied by a pronounced and sustained increase in cellular glucose uptake (Figure 3Q), glucose consumption (Figure 3R), and lactic acid production (Figure 3S). Furthermore, analysis of AMPK downstream effectors revealed that mTOR expression was significantly reduced in hs-P1 but subsequently increased (Figure 3J). Protein levels of Sirt1 and NAMPT (Figure 3K,L), along with cellular NAD^+^ levels (Figure 3P), peaked in hs-P1 and subsequently declined. In parallel, Western blot analysis showed that protein levels of PERK, p-PERK, and ATF4 were markedly increased in hs-P1 but progressively decreased thereafter (Figure 3G,M–O), indicating that heightened intracellular ROS triggered endoplasmic reticulum (ER) stress and the UPR [33]. Collectively, these findings outline a mechanistic cascade in which hyperosmotic stress triggers oxidative stress in HCEPCs, resulting in the decrease in mitochondrial membrane potential. This mitochondrial dysfunction further drives excessive production of ROS, thereby amplifying the initial oxidative stress. This, on one hand, disrupts cellular energy homeostasis, activating the AMPK/mTOR/NAMPT/Sirt1 signaling axis and triggering an energy stress response. On the other hand, it induces ER stress, activating the PERK/ATF4 signaling pathway and eliciting the UPR [34].

### 3.4. Autophagy, Particularly Mitophagy, in HCEPCs Are Activated by Oxidative Stress-Induced Energy Stress Response and UPR

**Figure 4:**
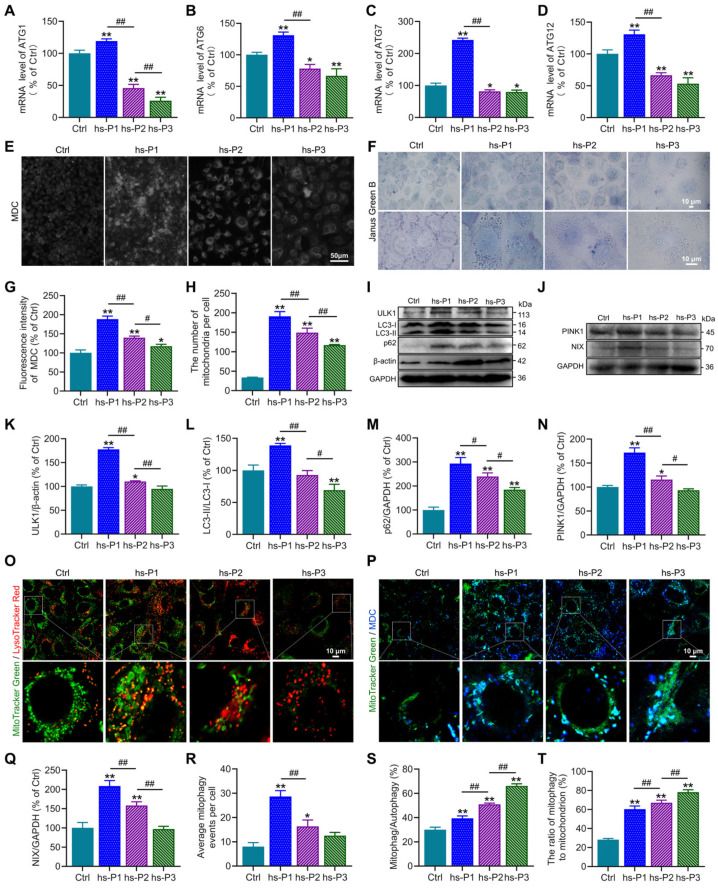
Hyperosmotic stress activates autophagy and mitophagy in HCEPCs to counterbalance cellular homeostasis. (**A**–**D**) Detection and statistical analysis of mRNA expression levels of ATF4 target genes *ATG1*, *ATG6*, *ATG7*, and *ATG12* in hypertonic-stressed HCEPCs. (**E**) Fluorescent images of MDC staining in hypertonic-stressed HCEPCs of different passages. (**F**) Images of Janus green B staining in hypertonic-stressed HCEPCs of different passages. (**G**) Statistical analysis of MDC staining in (**G**). (**H**) Statistical analysis of mitochondrial numbers in (**F**). (**I**) Western blot images of ULK1, LC3 and p62 in hypertonic-stressed HCEPCs of different passages. (**J**) Western blot images of PINK and NIX in hypertonic-stressed HCEPCs of different passages. (**K**–**M**) Optical density analysis of ULK1, LC3 and p62 immunoblots in (**I**). (**N**,**Q**) Optical density analysis of PINK and NIX immunoblots in (**J**). (**O**) Co-localization fluorescence images of MitoTracker Green/LysoTracker Red in hypertonic-stressed HCEPCs of different passages. Representative micrographs display mitochondria (green) and lysosomes (red). (**P**) Co-localization fluorescence images of MitoTracker Green/MDC in hypertonic-stressed HCEPCs of different passages. Representative micrographs display mitochondria (green) and autophagosomes (blue). (**R**) Statistical analysis of mitochondrial autophagy-positive points in (**O**). (**S**) Statistical analysis of the proportion of mitochondrial autophagy to total autophagy in (**P**). (**T**) Statistical analysis of the proportion of autophagic mitochondria to total mitochondria in (**P**). hs-P denotes the number of successive passages under 400 mOsm/L hyperosmotic medium. Data are expressed as mean ± SEM (*n* = 3). * p < 0.05, ** p < 0.01 vs. blank controls (Ctrl), and *^#^ p* < 0.01, *^##^ p* < 0.01 among different experimental groups.

Autophagy, a conserved cellular self-degradative process, plays pivotal roles in maintaining energy homeostasis and clearing misfolded proteins, protein aggregates, and damaged organelles [35]. Our protein analyses revealed elevated expression of ATF4, a master transcriptional regulator of autophagy-related genes, in hyperosmotically stressed HCEPCs. To functionally validate ATF4’s role, we quantified mRNA levels of key *ATGs* (*ATG1*, *ATG6*, *ATG7*, *ATG12*) using RT-PCR. Consistent with ATF4 protein dynamics, the mRNA levels of these genes peaked at hs-P1 and progressively declined thereafter (Figure 4A–D). Concurrently, the AMPK/mTOR/NAMPT/Sirt1 signaling axis, known to regulate autophagy activation, was dynamically modulated. We next assessed autophagic flux using MDC staining to label autophagosomes. Autophagy was significantly activated upon hyperosmotic stress, reaching maximal levels at hs-P1 before declining with successive passages (Figure 4E,G). This activation profile was corroborated by concordant trends in the protein expression of autophagy markers ULK1, LC3 and p62 (Figure 4I,K–M). These findings indicate that the energy crisis response and UPR triggered by oxidative stress promote autophagy activation and related gene transcription, likely as an adaptive mechanism to alleviate energy deficits and clear misfolded proteins [36]. Given that excessive ROS generation—a major incentive of intracellular dyshomeostasis under hyperosmotic stress—primarily originates from damaged mitochondria, we investigated the role of mitophagy, the selective autophagic clearance of mitochondria. Western blot analysis revealed that protein levels of PINK1 (a key protein of ubiquitin-dependent mitophagy) and NIX (a key protein of ubiquitin-independent mitophagy) peaked at hs-P1 and subsequently declined (Figure 4J,N,Q). Notably, total mitochondrial mass, assessed by Janus green B staining, was highest at hs-P1 and then decreased (Figure 4F,H), aligning with changes in mitophagic activity. Based on this findings, we hypothesize that mitophagy may exert a regulatory influence on mitochondrial biogenesis [37]. We performed co-staining with MitoTracker Green (mitochondria) and LysoTracker Red (lysosomes) observing mitophagy directly in comparison with total autophagosomes (MDC) to calculate the mitophagic rate in total autophagy. Hyperosmotic stress significantly increased the number of MitoTracker Green/LysoTracker Red double-positive puncta (indicating mitolysosome formation), which peaked at hs-P1 and declined thereafter (Figure 4O,R). Furthermore, the proportion of mitophagy relative to total autophagy and the ratio of mitochondria undergoing mitophagy significantly increased with stress exposure (Figure 4P,S,T). Collectively, these results delineate a coordinated adaptive response: Oxidative stress, on one hand, activates the AMPK signaling pathway via the energy crisis response, promoting autophagosome biogenesis. On the other hand, it induces the UPR, activating the PERK/ATF4 signaling axis to enhance transcription of autophagy genes. This dual activation significantly elevates overall autophagy levels to counteract cellular dyshomeostasis. Critically, as damaged mitochondria are the primary source of ROS, mitophagy is robustly upregulated—particularly during early stress (hs-P1)—facilitating mitochondrial quality control and renewal. However, the relentless accumulation of ROS under sustained hyperosmotic stress ultimately overwhelms these protective mechanisms, precipitating irreversible cellular damage and senescence.

### 3.5. Activation of Autophagy Attenuates Oxidative Stress by Enhancing Mitophagy in Hyperosmotically Stressed HCEPCs

**Figure 5:**
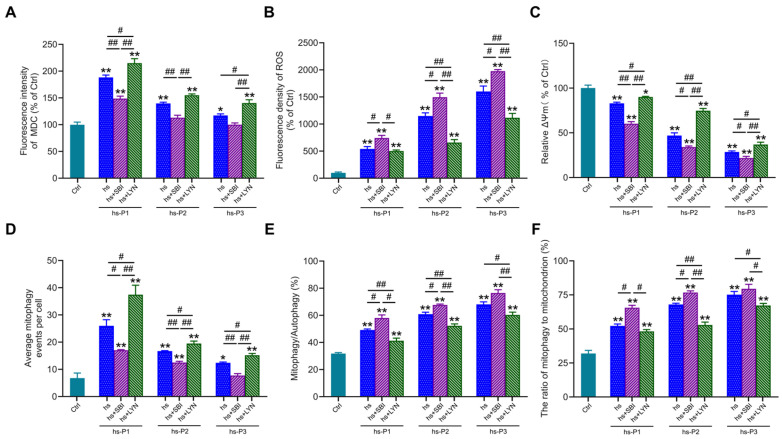
Activation of autophagy in response to hyperosmotic stress enhances mitophagic activity and ameliorates oxidative stress in HCEPCs. (**A**) Statistical analysis of MDC staining in hyperosmotic-stressed HCEPCs after treatment with autophagy inhibitors/activators.in Figure S2A. (**B**) Statistical analysis of ROS levels in hyperosmotic-stressed HCEPCs after treatment with autophagy inhibitors/activators.in Figure S2B. (**C**) Statistical analysis of mitochondrial membrane potential in hyperosmotic-stressed HCEPCs after treatment with autophagy inhibitors/activators. (**D**) Statistical analysis of mitochondrial autophagy-positive points in hyperosmotic-stressed HCEPCs after treatment with autophagy inhibitors/activators in Figure S2C. (**E**) Statistical analysis of the proportion of mitochondrial autophagy to total autophagy in hyperosmotic-stressed HCEPCs after treatment with autophagy inhibitors/activators in Figure S2D. (**F**) Statistical analysis of the proportion of autophagic mitochondria to total mitochondria in hyperosmotic-stressed HCEPCs after treatment with autophagy inhibitors/activators in Figure S2D. P denotes the number of successive passages. Data are expressed as mean ± SEM (*n* = 3). * p < 0.05, ** p < 0.01 vs. blank controls (Ctrl), and *^#^ p* < 0.01, *^##^ p* < 0.01 among different experimental groups.

To further elucidate the functional role of autophagy in hyperosmotic stress-induced HCEPC senescence, we employed the autophagy inhibitor SBI-0206965 and activator LYN-1604 to modulate autophagic activity. MDC staining (Figure S2A and Figure 5A) confirmed that SBI-0206965 effectively suppressed, while LYN-1604 robustly enhanced, autophagic flux in stressed HCEPCs (compared to the hs group alone). Concomitantly, SBI-0206965 treatment significantly exacerbated intracellular ROS levels across passages P1–P3 under hyperosmotic stress. Conversely, LYN-1604 markedly attenuated oxidative stress in all passages examined (Figure S2B and Figure 5B). To determine whether altered autophagy influences ROS levels by modulating mitochondrial integrity, ΔΨm was assessed via JC-1 staining. Strikingly, autophagy inhibition further potentiated the loss of ΔΨm induced by hyperosmotic stress. In contrast, autophagy activation effectively preserved ΔΨm, indicating reduced mitochondrial damage (Figure 5C). Subsequently, we evaluated mitophagic activity using MitoTracker Green/LysoTracker Red (Figure S2C) and MitoTracker Green/MDC (Figure S2D) co-localization assays. SBI-0206965 significantly inhibits autophagy, most of which is mitophagy (Figure 5D) while concurrently increasing both the proportion of mitophagy relative to total autophagy (Figure 5E), and the fraction of mitochondria undergoing mitophagy (Figure 5F). Conversely, LYN-1604 significantly enhances autophagy, most of which is mitophagy, while reducing the aforementioned mitophagy proportions. Collectively, these results demonstrate that pharmacological activation of autophagy using LYN-1604 significantly enhances mitophagic flux in hyperosmotically stressed HCEPCs, thereby attenuating ROS generation and alleviating cellular oxidative burden. Conversely, autophagy inhibition synergistically with hyperosmotic stress exacerbated mitochondrial damage and intensified the cellular oxidative stress response.

### 3.6. Mitophagy Activation Mitigates Energy Stress, DNA Damage, and Inflammation by Enhancing Mitophagy in HCEPCs Under Hyperosmotic Stress

**Figure 6:**
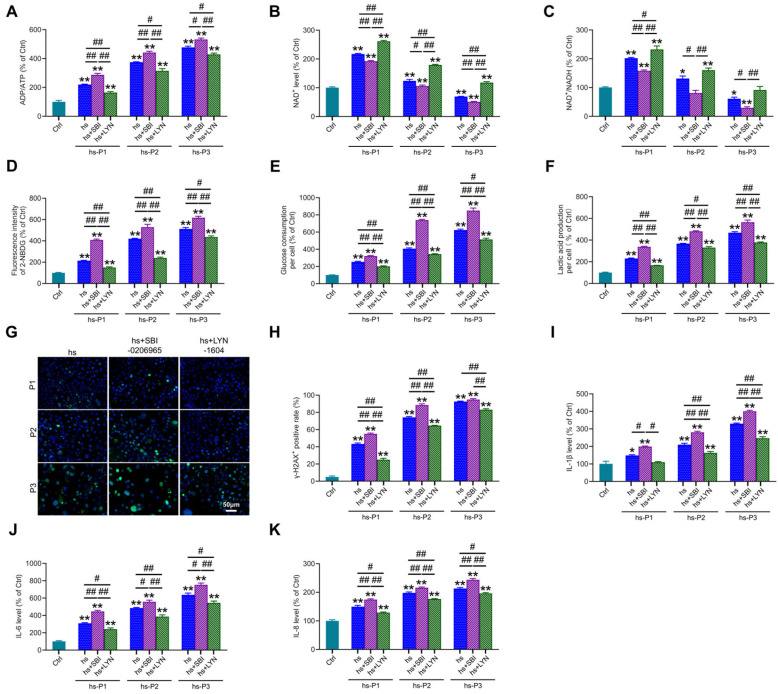
Autophagy activation alleviates energy crisis, DNA damage, and inflammatory response in hyperosmotic-stressed HCEPCs. (**A**) Detection of ATP/ADP levels in hypertonic-stressed HCEPCs after treatment with autophagy inhibitors/activators. (**B**) Detection of NAD^+^ levels in hypertonic-stressed HCEPCs after treatment with autophagy inhibitors/activators. (**C**) Detection of the NAD^+^/NADH ratio in hyperosmotic-stressed HCEPCs after treatment with autophagy inhibitors/activators. (**D**) Detection of cellular glucose uptake levels in hyperosmotic-stressed HCEPCs after treatment with autophagy inhibitors/activators. (**E**) Detection of glucose consumption levels in hyperosmotic-stressed HCEPCs after treatment with autophagy inhibitors/activators. (**F**) Detection of lactic acid production levels in HCEPCs subjected to hyperosmolar stress after treatment with autophagy inhibitors/activators. (**G**,**H**) Immunofluorescence staining images and statistical analysis of γ-H2AX in hyperosmotic-stressed HCEPCs treated with autophagy inhibitors/activators. Representative micrographs display γ-H2AX -positive cells (green) and total cells (blue). (**I**–**K**) Detection of IL-1β, IL-6, and IL-8 secretion levels in hyperosmotic-stressed HCEPCs treated with autophagy inhibitors/activators. P denotes the number of successive passages. Data are expressed as mean ± SEM (*n* = 3). * p < 0.05, ** p < 0.01 vs. blank controls (Ctrl), and *^#^ p* < 0.01, *^##^ p* < 0.01 among different experimental groups.

We next assessed key energy parameters—ADP/ATP ratio (Figure 6A), NAD^+^ levels (Figure 6B), and NAD^+^/NADH ratio (Figure 6C)—in autophagy-modulated HCEPCs under hyperosmotic stress. This evaluated whether autophagy-dependent changes in mitochondrial integrity and oxidative burden influence the cellular energy crisis. Notably, compared to the hyperosmotic stress (hs) group alone, autophagy inhibition significantly exacerbated the energy deficit, manifested by (i) elevated ADP/ATP ratio, (ii) reduced NAD^+^ levels, and (iii) diminished NAD^+^/NADH ratio. Conversely, mitophagy activation markedly improved cellular energetics, demonstrating: (i) decreased ADP/ATP ratio, (ii) increased NAD^+^ levels, and (iii) enhanced NAD^+^/NADH ratio. Concomitantly, autophagy inhibition further amplified hyperosmotic stress-induced glycolytic activity, evidenced by significant increases in cellular glucose uptake (Figure 6D), glucose consumption (Figure 6E) and lactic acid production (Figure 6F). Autophagy activation effectively suppressed this hyperosmotic stress-induced glycolytic shift. Collectively, these metabolic findings indicate that impaired mitophagy aggravates the energy crisis under hyperosmotic stress, whereas enhanced mitophagy alleviates oxidative stress and energy deficits by improving mitochondrial biogenesis and bioenergetic output. Given that ROS overproduction under hyperosmotic stress induces DNA damage, and cytosolic accumulation of fragmented DNA may potentiate inflammatory responses, we examined DNA integrity and inflammation. Mitophagy inhibition potentiated DNA damage, marked by increased γ-H2AX-positive cell (Figure 6G,H), and exacerbated inflammatory cytokine release (IL-1β: Figure 6I; IL-6: Figure 6J; IL-8: Figure 6K). Strikingly, mitophagy activation significantly attenuated both DNA damage and inflammatory cytokine secretion. These results delineate a dual protective mechanism: Enhancing autophagy, particularly mitophagy, in hyperosmotically stressed HCEPCs, on one hand, promotes mitochondrial renewal and enhances bioenergetic capacity, thereby alleviating the energy crisis. On the other hand, it suppresses ROS generation, mitigating DNA damage and subsequent inflammatory cascades.

### 3.7. Mitophagy Activation Attenuates Cellular Senescence in Hyperosmotically Stressed HCEPCs

**Figure 7:**
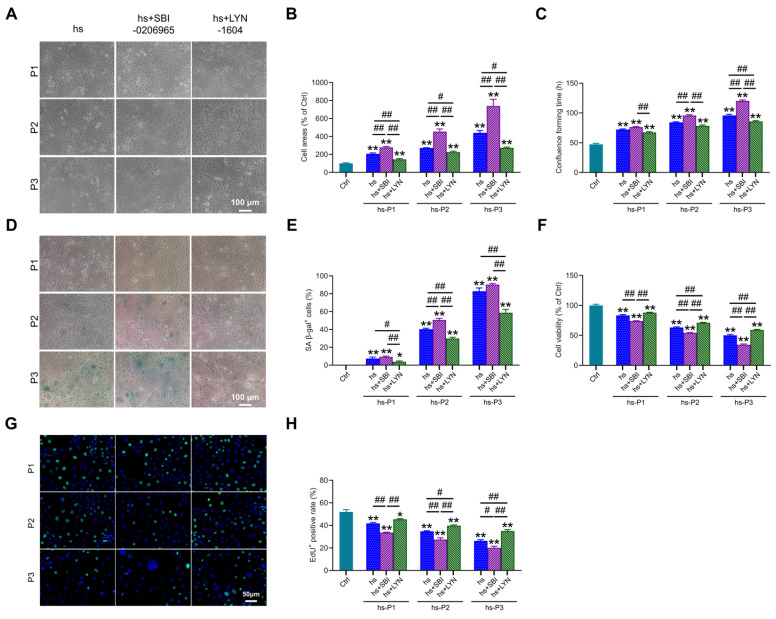
Autophagy activation delays hyperosmotic stress-induced senescence in HCEPCs. (**A**) Light microscope images of HCEPCs subjected to hyperosmotic stress after treatment with autophagy inhibitors/activators. (**B**) Statistical analysis of cell area in (**A**). (**C**) Statistical analysis of confluence time of HCEPCs subjected to hyperosmotic stress after treatment with autophagy inhibitors/activators. (**D**) SA-β-gal staining images of HCEPCs subjected to hyperosmotic stress after treatment with autophagy inhibitors/activators. (**E**) Statistical analysis of the percentage of SA-β-gal-positive cells in (**D**). (**F**) Cell viability assay of HCEPCs subjected to hyperosmotic stress after treatment with autophagy inhibitors/activators. (**G**,**H**) EdU staining images and statistical analysis of HCEPCs subjected to hyperosmotic stress after treatment with autophagy inhibitors/activators. Representative micrographs display EdU-positive cells (green) and total cells (blue). P denotes the number of successive passages. Data are expressed as mean ± SEM (*n* = 3). * p < 0.05, ** p < 0.01 vs. blank controls (Ctrl), and *^#^ p* < 0.01, *^##^ p* < 0.01 among different experimental groups.

Building upon the established role of autophagy in regulating multiple stress-response pathways, we investigated its ultimate impact on senescence hallmarks. Notably, compared to the hs group, autophagy (particularly mitophagy) inhibition further exacerbated senescence-associated phenotypes, manifested by: (i) increased cell surface area (Figure 7A,B), (ii) prolonged time to reach confluency (Figure 7C), and (iii) elevated proportion of SA-β-gal-positive cells (Figure 7D,E). Conversely, pharmacological activation of mitophagy significantly mitigated these alterations. Concomitantly, mitophagy inhibition markedly reduced cell viability (Figure 7F) and proliferative capacity (Figure 7G,H), whereas mitophagy enhancement robustly preserved both parameters relative to the hs group. Collectively, these findings demonstrate that augmenting mitophagy following hyperosmotic stress enhances mitochondrial quality control, thereby attenuating oxidative burden, energy crisis, DNA damage, and inflammatory cascades. This coordinated mitigation significantly decelerates the progression of HCEPC senescence. In stark contrast, mitophagy impairment profoundly accelerates the senescence trajectory.

## 4. Discussion

Current clinical management strategies of DED—including artificial tears, anti-inflammatory agents (e.g., cyclosporine A, corticosteroids), and physical therapies (e.g., thermal pulsation, meibomian gland massage)—mainly alleviate symptoms but often lead to recurrence upon discontinuation [38]. The lack of lasting efficacy of these therapies stems from an incomplete understanding of their underlying mechanisms, hampering the development of targeted therapies. Therefore, clarifying DED pathogenesis and identifying novel therapeutic targets are of critical scientific and clinical importance.

The tear hyperosmolarity induced by DED triggers the release of pro-inflammatory factors such as IL-1, IL-6, and IL-8 exacerbates ocular surface inflammation [39]. This process not only produces the symptoms and tissue damage associated with DED but also further elevates tear osmolarity, thereby creating a self-perpetuating vicious cycle [40]. Studies have elucidated that hyperosmotic stress induces corneal epithelial defects and thickening in mice [41]. In parallel, extensive in vitro investigations have revealed that hyperosmotic exposure elicits inflammatory responses in HCEPCs, which can even lead to cell death [42,43,44]. Meanwhile, a growing number of studies have revealed that oxidative stress, triggered by hyperosmotic stress, is a key mediator of the inflammatory response and ocular surface damage in dry eye disease [45,46,47,48,49], thereby promoting corneal epithelial cell senescence [50,51,52,53,54].

Autophagy is a highly conserved intracellular degradation process in eukaryotic cells that facilitates the lysosome-mediated breakdown of misfolded proteins and damaged organelles [55]. The sequential stages of autophagy encompass initiation (via ULK1 complex assembly), autophagosome formation (characterized by the conversion of LC3-I to LC3-II), and ultimately, cargo degradation following autophagosome-lysosome fusion (a process mediated by the adaptor protein p62) [56]. Under physiological conditions, autophagy supports cellular renewal and protein quality control; during nutrient deprivation [57], it serves as a survival mechanism by recycling non-essential components [58]; under stress, it clears damaged proteins and organelles to mitigate cytotoxicity [59].

Clinical studies have reported elevated autophagy markers in the tear film and conjunctival epithelium of DED patients [60], and evidence from studies in vitro consistently demonstrates autophagy activation in cellular models of dry eye or hyperosmotic stress [61]. Moderate autophagy activation protects cells by clearing damaged mitochondria, reducing oxidative stress, and suppressing inflammation [62,63,64]. However, excessive oxidative stress can lead to autophagosome accumulation and impaired autophagic flux, exacerbating corneal epithelial injury [65]. Recent research has further elucidated the molecular mechanisms underlying autophagy-mediated protection in DED. Evodiamine alleviates oxidative stress and promotes corneal epithelial repair in dry eye disease by activating autophagy through the p53/mTOR pathway [66], while Salidroside reduces oxidative damage in dry eye by enhancing autophagy via the AMPK-Sirt1/Nrf2 axis [67]. Therefore, targeted and controlled activation of autophagy represents a promising therapeutic strategy for DED.

The present study elucidates the intricate molecular cascade through which chronic hyperosmotic stress drives HCEPC senescence, a pivotal mechanism in DED pathogenesis. We establish that sustained exposure to 400 mOsm/L—mirroring peak clinical tear hyperosmolarity—induces a progressive senescent phenotype characterized by increased cell area, proliferative arrest, declined cell viability, DNA damage, nuclear lamina disassembly, and amplified SASP factors secretion. Notably, Lamin B1 disruption signifies irreversible nuclear envelope damage, a recognized commitment point to senescence [68]. DNA damage activates the cGAS-STING-NF-κB signaling pathway, which subsequently triggers the secretion of SASP factors and a pro-inflammatory response. This cascade ultimately exacerbates cellular senescence and perpetuates ocular surface inflammation in DED [69]. Crucially, our research results reveal that this process is orchestrated through mitochondrial dysfunction-induced oxidative stress and bioenergetic crisis, with autophagy (especially mitophagy) acting as a critical adaptive nexus determining cellular fate.

We demonstrate that hyperosmolarity induces a metabolic vicious cycle: hyperosmotic stress leads to intracellular environmental disorder, intracellular environmental disorder impairs the functions of organelles such as mitochondria, ER. The dysfunctional mitochondria produce excess ROS exacerbating oxidative damage to further compromise ATP synthesis resulting in an increase in ADP/ATP ratios. The excess ROS also exert oxidative stress on ER leading to misfolded protein accumulation synergizes with oxidative injury to overwhelm cellular homeostasis. In response to the oxidative stress on mitochondria and ER, the increased ADP/ATP ratios activate AMPK/mTOR/NAMPT/Sirt1 axis to increase conversion of LC3I to LC3II and the misfolded protein accumulation activates UPR of PERK/ATF4 pathways to enhance the expression of *ATGs* (*ATG1*, *ATG6*, etc.). Thus, both the increased conversion of LC3I to LC3II and upregulated *ATGs* expression trigger autophagy to highlight a compensatory effort of restoring metabolic balance. However, these protective adaptive responses eventually succumb to persistent hyperosmotic stress, resulting in the collapse of cellular homeostasis and thereby triggering irreversible DNA damage (γ-H2AX accumulation) and SASP factors secretion (IL-1β, IL-6, IL-8), mirroring the chronic inflammatory milieu observed in DED [70].

Our data position autophagy as a pivotal regulator of HCEPCs survival under hyperosmotic stress. In the early of hypertonic stress (hs-P1), the activation of autophagy, marked by elevated LC3-II/LC3-I ratios, ULK1 and p62 level, and ATF4-driven transcription, reflects its dual role in mitigating both energy crises and proteotoxic stress. However, sustained stress exhausts autophagic capacity, permitting ROS accumulation and irreversible damage. Critically, mitophagy—the selective clearance of damaged mitochondria—emerges as a cornerstone of this response. The peak expression of PINK1 and NIX in early passages, coupled with increased mitophagic flux (MitoTracker/LysoTracker co-localization), demonstrates that autophagy prioritizes mitochondrial quality control to curb ROS overproduction. This is corroborated by the pharmacological experiments: autophagy inhibition (SBI-0206965) exacerbates mitochondrial depolarization and oxidative burden, while its activation (LYN-1604) preserves ΔΨm and reduces ROS, thereby attenuating downstream DNA damage and SASP factors secretion. The disproportionate impact of autophagy on mitophagy (the proportion of mitophagy relative to total autophagy) underscores mitochondrial quality control as the primary therapeutic node.

The reciprocal crosstalk between autophagy and energy metabolism crucially amplifies its cytoprotective effects. Mitophagy activation enhances mitochondrial biogenesis to improve aerobic respiration, which not only alleviates ADP/ATP imbalance and replenishes NAD^+^ levels but also strikingly suppresses hyperosmotic stress-induced glycolytic hyperactivity—evidenced by reduced glucose uptake/consumption and diminished lactate production. This metabolic reprogramming resolves bioenergetic crises, thereby preserving cellular viability and proliferative capacity, ultimately contributing to significant attenuation of senescence progression in hyperosmotically stressed HCEPCs.

**Figure 8:**
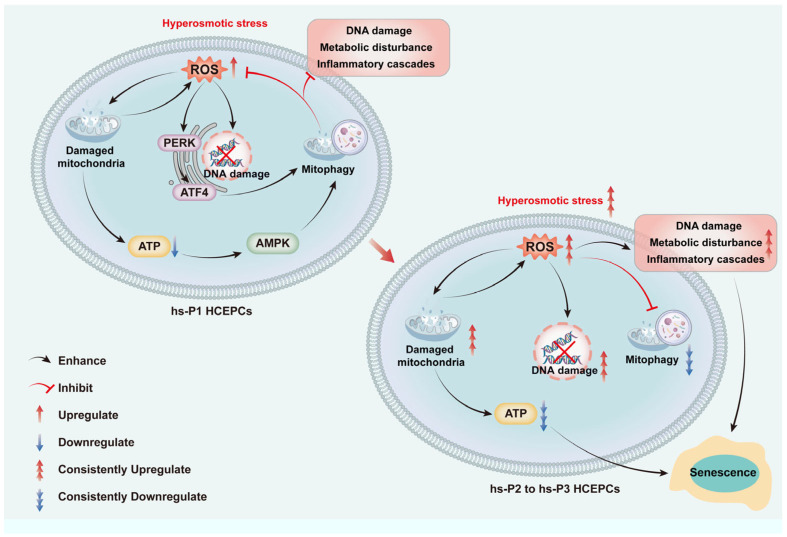
Graphical mechanism of HCEC senescence induced by hyperosmolarity through oxidative stress-dependent DNA damage, metabolic disturbance and mitophagy decline.

Collectively, we propose a sequential model (as shown in Figure 8): Hyperosmotic stress compromises mitochondrial integrity → ROS overproduction induces oxidative damage, energy deficit (activating AMPK), and ER stress (activating PERK/ATF4) → The AMPK and ATF4 pathways converge to upregulate general autophagy most of which are mitophagy → Efficient mitophagy clears damaged mitochondria, curbing ROS at its source and preserving energy output → This attenuates DNA damage, metabolic disturbance, and inflammatory cascades → Cellular homeostasis is temporarily preserved, delaying senescence. However, sustained hyperosmotic stress eventually overwhelms autophagic capacity, leading to mitochondrial collapse, metabolic disturbance, and senescence execution.

Current DED therapies primarily target surface inflammation or tear replacement [71], yet our data suggest that restoring autophagy could address the root causes of epithelial dysfunction. By enhancing mitochondrial turnover and suppressing oxidative stress, autophagy modulation may offer a dual benefit: preserving HCEPCs integrity and dampening the chronic inflammatory loop. This aligns with emerging evidence linking autophagy dysregulation to age-related ocular surface diseases, positioning autophagy activators (e.g., LYN-1604) as promising candidates for adjunctive therapies. However, several questions remain. For instance, the long-term efficacy of autophagy modulation in vivo, the potential off-target effects of pharmacological agents, and the interplay between autophagy and other stress-responsive pathways warrant further investigation.

While this study provides mechanistic insights, it is limited to in vitro models. Future research should validate these findings in ex vivo human corneal tissues and in vivo DED models. Longitudinal studies assessing autophagy dynamics in patient-derived HCEPCs could bridge the gap between bench and bedside. Furthermore, high-throughput screening for autophagy-modulating compounds with ocular bioavailability would accelerate therapeutic development.

## 5. Conclusions

In conclusion, this study demonstrates that hyperosmolarity-induced oxidative stress is a central driver of HCEPC senescence in dry eye disease. Hyperosmotic stress compromises mitochondrial dysfunction, which triggers concurrent DNA damage and bioenergetic crisis. A critical finding is the progressive failure of compensatory mitophagy under sustained stress, leading to the accumulation of damaged mitochondria, which perpetuates oxidative injury and amplifies inflammatory signaling. Our results establish that the potentiation of mitophagy serves as a key protective mechanism, highlighting its potential as a therapeutic target to decelerate corneal epithelial senescence and disrupt the pathogenic cycle of DED.

## Data Availability

The data presented in this study are available from the corresponding author upon reasonable request.

## Supplementary Materials

The following supporting information can be downloaded at https://www.mdpi.com/article/10.3390/antiox14111381/s1, Figure S1: Fluorescent images of JC-1 staining in hypertonic-stressed HCEPCs of different passages. Figure S2: Effects of autophagy modulators on autophagy, ROS production, and mitophagy in hyperosmotic-stressed HCEPCs.

## Abbreviations

The following abbreviations are used in this manuscript:

2-NBDG	2-(N-(7-Nitrobenz-2-oxa-1,3-diazol-4-yl)Amino)-2-Deoxyglucose
AMPK	adenosine monophosphate-activated protein kinase
ATG	autophagy-related gene
CCK-8	Cell Counting Kit-8
cGAS	cyclic GMP-AMP synthase
DAPI	4′,6-diamidino-2-phenylindole
DED	Dry eye disease
DMEM	Dulbecco’s Modified Eagle Medium
ECL	enhanced chemiluminescence
EdU	5-ethynyl-2′-deoxyuridine
ELISA	enzyme-linked immunosorbent assay
ER	endoplasmic reticulum
FBS	fetal bovine serum
GAPDH	glyceraldehyde-3-phosphate dehydrogenase
HCEPC	human corneal epithelial cells
IL	Interleukin
LC3	microtubule-associated protein 1 light chain 3
MDC	monodansylcadaverine
MMPs	matrix metalloproteinases
mTOR	mammalian target of rapamycin complex
NAD	nicotinamide adenine dinucleotide
NAMPT	nicotinamide phosphoribosyl transferase
NF-κB	nuclear factor kappa-light-chain-enhancer of activated B cells
NIX	Nip3-like protein X
PBS	Phosphate-buffered saline
PBST	phosphate-buffered saline with Tween 20
PERK	protein kinase R like endoplasmic reticulum kinase
PINK1	PTEN-induced kinase 1
PVDF	polyvinylidene difluoride
RIPA	radio-immunoprecipitation assay lysis buffer
ROS	reactive oxygen species
RT-qPCR	real-time reverse transcription polymerase chain reaction
SASP	senescence-associated secretory phenotype
SA-β-gal	senescence-associated-β-galactosidase
SDS-PAGE	sodium dodecyl sulfate-polyacrylamide gel electrophoresis
SEM	standard error of the mean
Sirt1	silent information regulator factor 2-related enzyme 1
STING	stimulator of interferon genes
TNF-α	tumor necrosis factor-α
ULK1	Unc-51 like autophagy activating kinase 1
UPR	unfolded protein response
