# Supplementary material for: Hyperosmolarity-Induced Oxidative Stress Leads to Senescence in Human Corneal Epithelial Cells (HCEPC) via DNA Damage, Metabolic Disturbance and Mitophagy Decline
Source: Antioxidants (Basel). 2025 Nov 19;14(11):1381. doi: 10.3390/antiox14111381 (PMC12649220; doi:10.3390/antiox14111381)
Supplement: Supplementary file 1 [file antioxidants-14-01381-s001.zip › antioxidants-3961024-supplementary.pdf]

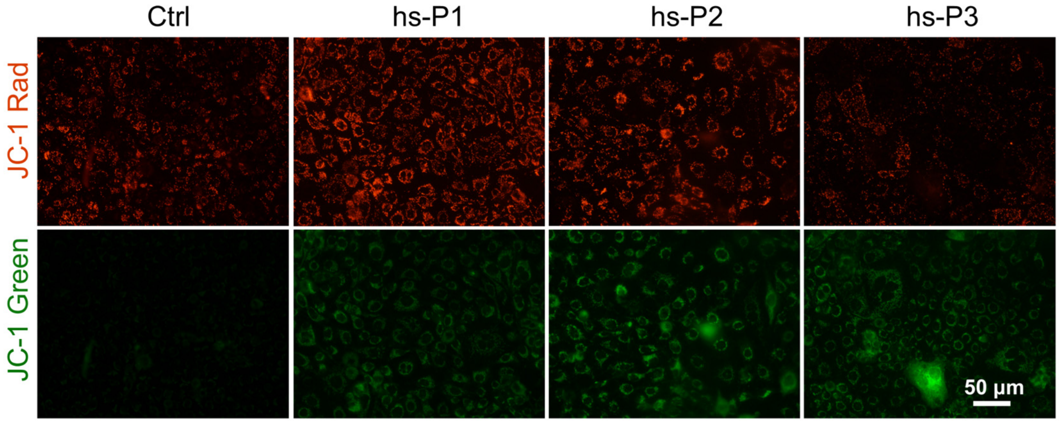

Figure S1. Fluorescent images of JC-1 staining in hypertonic-stressed HCEPCs of different passages. Representative micrographs display aggregated JC-1 (red) and monomeric JC-1 (green).

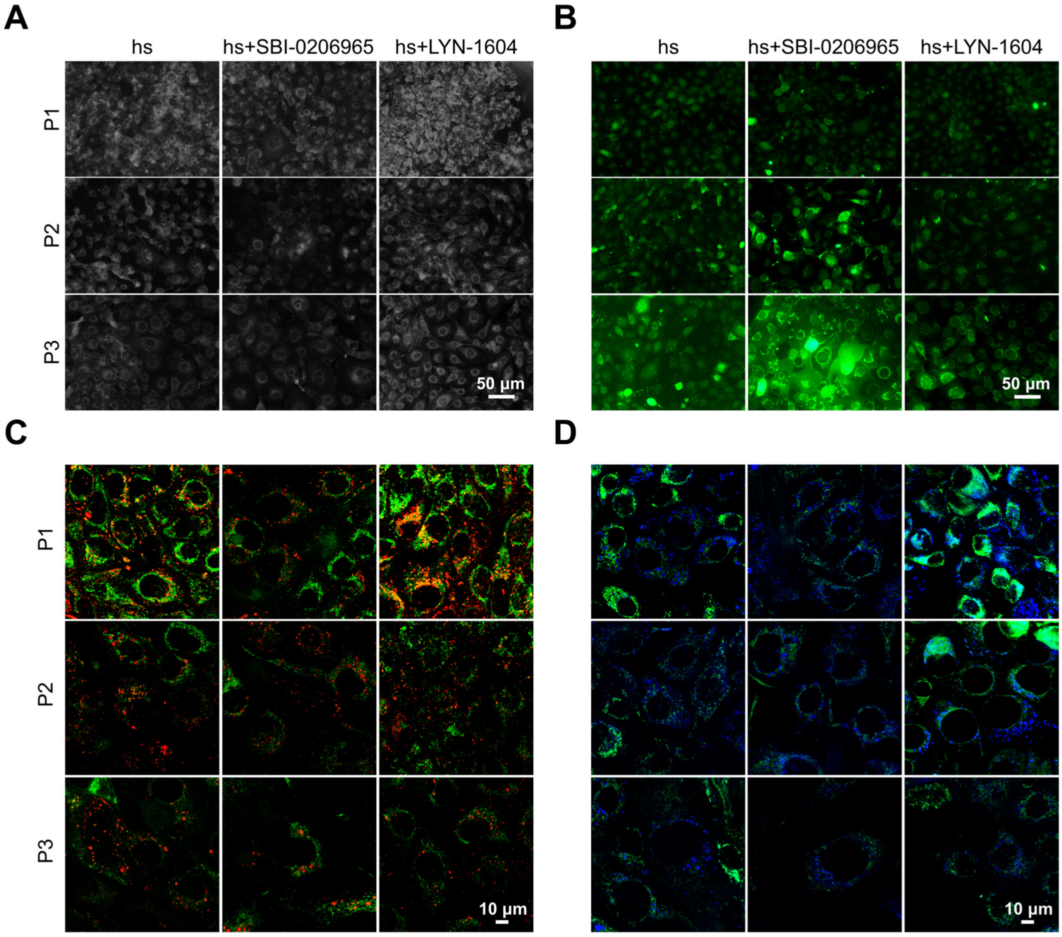

Figure S2. Effects of autophagy modulators on autophagy, ROS production, and mitophagy in hyperosmotic-stressed HCEPCs. **(A)** MDC staining images in hyperosmotic-stressed HCEPCs treated with autophagy inhibitors/activators. **(B)** ROS staining images in hyperosmotic-stressed HCEPCs treated with autophagy inhibitors/activators. **(C)** Co-localization fluorescence images of MitoTracker Green/LysoTracker Red in hyperosmotic-stressed HCEPCs treated with autophagy inhibitors/activators. Representative micrographs display mitochondria (green) and lysosomes (red). **(D)** Co-localization fluorescence images of MitoTracker Green/MDC in hyperosmotic-stressed HCEPCs treated with autophagy inhibitors/activators. Representative micrographs display mitochondria (green) and autophagosomes (blue).
